# Unraveling the Molecular Mechanism of Bider Marking Formation in Dun Mongolian Horses Through Transcriptome Sequencing

**DOI:** 10.3390/ani16081145

**Published:** 2026-04-09

**Authors:** Tana An, Manglai Dugarjaviin

**Affiliations:** Inner Mongolia Key Laboratory of Equine Science Research and Technology Innovation, Inner Mongolia Agricultural University, Hohhot 010018, China; 15848154479@163.com

**Keywords:** Bider marking, Mongolian horse, transcriptome sequencing, differentially expressed genes, pigmentation

## Abstract

(1) Background: The “Bider” marking refers to the symmetrical black stripes distributed on the shoulder blades of Dun Mongolian horses, representing an ancestral trait of significant genetic value. However, the molecular mechanisms underlying its formation remain unclear. This study aims to elucidate the molecular basis of these markings by comparing transcriptomic differences in skin tissues from variously pigmented areas of Mongolian horses’ “Bider” patterns. (2) Methods: Using three Dun Mongolian horses as subjects, skin tissue samples were collected from their shoulders (dark-marked and light-marked areas), dorsal midline, and croup regions for transcriptome sequencing. Differentially expressed genes were identified based on sequencing data, followed by Gene Ontology (GO) functional annotation and Kyoto Encyclopedia of Genes and Genomes (KEGG) pathway enrichment analysis. Key findings were validated through quantitative reverse transcription polymerase chain reaction (qRT-PCR). (3) Results: The sequencing yielded approximately 893 million high-quality clean reads, with an overall alignment rate exceeding 96%. A total of 140 to 775 differentially expressed genes were identified. GO enrichment analysis revealed that these genes were significantly enriched in biological processes related to pigment metabolism, skin and hair follicle development, signal transduction (including calcium and cyclic guanosine monophosphate (cGMP) signaling), and immune regulation. KEGG analysis further indicated that multiple pathways closely associated with pigment regulation, including the calcium signaling pathway, tyrosine metabolism, cyclic adenosine monophosphate (cAMP) signaling pathway, and melanoma pathway, were significantly enriched across different tissue comparison groups, suggesting their potential key roles in coat color phenotype formation. The reliability of the sequencing data was corroborated by the results of qRT-PCR validation. (4) Conclusions: This study conducted a transcriptome analysis of skin samples from various pigmented regions of the Dun Mongolian horse’s Bider marking, revealing that the formation of this marking is associated with the differential expression of numerous genes and is co-regulated by multiple pigment-related signaling pathways.

## Simple Summary

The black markings on the shoulders of Mongolian horses, also known as “Bider markings,” represent a unique genetic characteristic of this breed. However, the mechanisms underlying the formation of these markings remain unclear. This study investigates this enigma by analyzing gene activity in the shoulder markings and other control regions of horse skin. Our findings reveal significant differences in the activity levels of numerous genes between skin areas of varying colors. These genes are predominantly associated with pigment production, skin development, and essential intracellular signaling pathways, such as calcium signaling and cAMP signaling. This suggests that the formation of Bider markings is not governed by a single gene but rather results from the precise coordination among a series of genes and signaling pathways. Our work unveils, for the first time, the complex molecular network underlying this unique coat pattern, which not only aids in the scientific conservation and utilization of the valuable genetic resources of Mongolian horses but also provides new insights into the mechanisms underlying the formation of complex color patterns in animals.

## 1. Introduction

**Figure 1:**
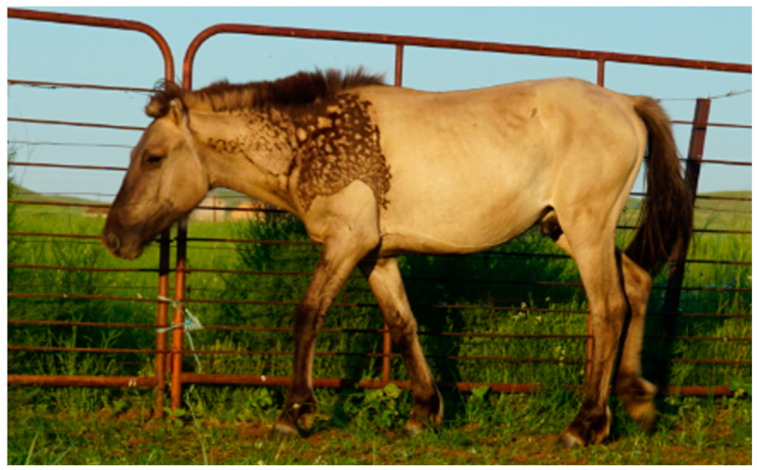
Bider marking of the Wuzhumuqin horse [4].

The Mongolian horse is an ancient breed of considerable genetic value [1,2]. The distinctive “Bider marking” of the Wuzhumuqin [3] horse stands out as particularly remarkable [4]. This unique coat pattern features irregular black spots symmetrically distributed across the shoulder area of a dun-colored coat [5,6] (Figure 1).

The Bider marking not only possesses unique aesthetic value but may also be closely associated with the survival adaptability of Mongolian horses in harsh environments [6]. However, the molecular mechanisms underlying its formation remain unclear to date. Therefore, elucidating these molecular mechanisms is crucial for understanding the spatial regulation principles of pigmentation in equine species, as well as for the conservation and sustainable utilization of the valuable genetic resource represented by Mongolian horses.

Coat color, a prominent phenotypic trait on animal surfaces, is regulated by both genetic factors and environmental conditions [7,8]. Mutations in the TBX3 gene have been closely associated with the formation of the dun phenotype in dun horse populations [9]. However, the distinctive pigmentation pattern known as the “Bider” marking typically appears on three different types of dun coat colors: yellow dun, red dun, and grullo [10]. Currently, there is a lack of systematic research on the region-specific pigmentation distribution mechanism of the Bider marking, particularly regarding the regulatory network of gene expression differences between marked and unmarked areas.

In recent years, the development of high-throughput sequencing technologies has provided powerful tools for systematically deciphering the genetic basis of complex traits [11,12]. Transcriptome sequencing can comprehensively reveal gene expression profiles of specific tissues or cells under particular conditions and has been successfully applied to the study of the mechanisms of coat color formation in various animals [13,14,15,16], including Rex rabbits [17], horses [18,19], cattle [20], sheep [21], mice [22], and pandas [23]. By comparing skin transcriptomes from different coat color regions or developmental stages, key differentially expressed genes and signaling pathways can be identified [24]. However, molecular-level research on the unique Bider marking trait remains notably scarce.

This study focuses on the dun Mongolian horse with typical Bider markings, hypothesizing that the formation of these markings results from the differential expression of key pigmentation genes between marked and unmarked areas. To test this hypothesis, we collected skin tissue samples from four distinct regions of the Bider horses (dark-marked area on the shoulder, light-marked area on the shoulder, dorsal midline, and croup) for transcriptome sequencing, aiming to achieve the following research objectives: (1) systematically compare gene expression profile differences among skin tissues from different regions; (2) identify key differentially expressed genes and signaling pathways associated with Bider marking formation; and (3) experimentally validate the expression patterns of key candidate genes. The research findings will elucidate the transcriptional regulatory mechanisms underlying the formation of special coat color patterns in Mongolian horses, providing a theoretical basis for the conservation and innovative utilization of equine genetic resources, as well as for studies on spatial-specific pigmentation in mammals.

## 2. Materials and Methods

### 2.1. Sample Collection and Ethical Statement

Skin tissue samples were collected from three healthy adult dun Wuzhumuqin Mongolian horses from Inner Mongolia (an 11-year-old female, 4-year-old female, and 6-year-old male), which exhibited typical Bider markings. Among them, the skin tissue samples came from the same three Bider-marked horses mentioned in [4]. The main reason is that the availability of Bider-marked horses, a valuable genetic resource, is very limited. To maximize the scientific value of these precious samples, we subdivided the collected tissue samples for different experimental analyses aimed at comprehensively elucidating the mechanism of Bider mark formation. These studies were conducted sequentially, focusing on different aspects, thus resulting in independent publications. The collection of skin tissue samples was completed in July 2023. All animal experimental procedures conducted in this study adhered to the protocol approved by the Experimental Animal Welfare and Ethics Committee of Inner Mongolia Agricultural University (Approval No.: NND2023085). Sampling was performed under combined anesthesia, with sedation and analgesia achieved through intravenous injection of xylazine (0.01–0.02 mg/kg) and butorphanol (0.02–0.04 mg/kg) to minimize discomfort for the animals [18]. Skin tissue samples measuring 1 × 1 cm were collected from four designated sites using a skin biopsy puncture technique: (1) the dark-marked area on the shoulder (BIDC); (2) the light-marked area on the shoulder (BILC); (3) the dorsal midline (BID); and (4) the croup (BIC). Three biological replicates were established for each site, resulting in a total of 12 samples. The samples were immediately placed in RNAlater preservation solution and stored in an ultra-low temperature freezer at −80 °C until RNA extraction.

### 2.2. RNA Extraction, Library Construction and Sequencing

Total RNA was extracted from approximately 30 mg of skin tissue samples. The specific procedures were as follows: the tissue samples were rapidly ground into a powder in liquid nitrogen, followed by total RNA extraction using TRIzol reagent (Invitrogen, Carlsbad, CA, USA) according to the manufacturer’s instructions. The integrity and quality of RNA were assessed by measuring the RNA Integrity Number (RIN) using an Agilent 2100 Bioanalyzer (Agilent Technologies, Santa Clara, CA, USA), while the concentration and purity (OD260/280 ratio) were determined using a NanoDrop One spectrophotometer (Thermo Scientific, Waltham, MA, USA). Samples with RIN > 5 and OD260/280 ratios between 1.9 and 2.2 were used for subsequent library preparation. For qualified RNA samples, 2 μg of total RNA was taken, and mRNA was enriched using magnetic beads with Oligo(dT). The mRNA was fragmented to approximately 300 bp under high-temperature ion conditions. Using the fragmented mRNA as a template, the first strand of cDNA was synthesized with random hexamer primers and M-MLV reverse transcriptase (Thermo Scientific, USA). Subsequently, the second strand of cDNA was synthesized using DNA polymerase I and RNase H. The double-stranded cDNA underwent end repair, A-tailing, and ligation with Illumina sequencing adapters. Library fragments were enriched by PCR amplification using specific primers, targeting an insert size of approximately 450 bp. The constructed library was quality-checked using the Agilent 2100 Bioanalyzer. Qualified libraries were precisely quantified for effective concentration by qPCR. Based on the effective concentrations of each library and the target sequencing data volume, libraries with different indexes were mixed proportionally. The pooled libraries were diluted to 2 nM and denatured into single strands, followed by paired-end sequencing (2 × 150 bp) on the Illumina NovaSeq 6000 platform (Annoroad Gene Technology (Beijing) Co., Ltd., Beijing, China) [25,26].

### 2.3. Sequencing Data Preprocessing and Differential Expression Analysis

The raw sequencing data were stored in FASTQ format, and the quality of the raw data was assessed using FastQC (v0.11.9). Trimmomatic (v0.39) was employed to remove adapter sequences and low-quality bases, with a quality threshold of Q < 20, resulting in high-quality clean reads. The Q30 and GC content of the clean reads were calculated to ensure that the data quality met the requirements for subsequent analysis. The clean reads were then aligned to the horse reference genome (EquCab3.0, downloaded from the ENSEMBL database) using HISAT2 (v2.1.0). Gene expression levels were estimated using StringTie (v2.1.4), with FPKM (Fragments Per Kilobase of transcript per Million mapped reads) serving as the normalized value for gene expression quantification. The formula for calculating FPKM is: FPKM = (10^3^ × F)/(N × L/10^6^), where F represents the number of fragments mapped to the gene, N denotes the total number of mapped fragments, and L indicates the gene length. Differential expression analysis was performed using DESeq2 (v1.20.0), with thresholds of |log2(Fold Change)| ≥ 1 and adjusted q-value (padj) < 0.05 to identify differentially expressed genes [27].

### 2.4. GO Functional and KEGG Pathway Enrichment Analysis

In the Gene Ontology (GO) enrichment analysis of differentially expressed genes, the number of genes associated with each GO term is initially calculated. Subsequently, the hypergeometric test is employed to identify GO terms that are significantly enriched in the differentially expressed genes in comparison to the entire genome background. The calculated *p*-values are adjusted, and a threshold of q ≤ 0.05 is established. GO terms that meet this criterion are classified as significantly enriched within the differentially expressed genes. Through GO functional enrichment analysis, the primary biological functions associated with the differentially expressed genes can be elucidated. The Kyoto Encyclopedia of Genes and Genomes (KEGG) serves as a comprehensive database for genomic decoding. Given a complete set of genes on a chromosome, it can predict the roles of protein interaction networks in various cellular activities. Hypergeometric tests are applied to each pathway in KEGG for enrichment analysis to identify significantly enriched pathways among the differentially expressed genes. Following integration, both GO and KEGG enrichment analyses utilize hypergeometric tests to identify significantly enriched functions or pathways within the differentially expressed genes, thereby elucidating their biological significance [28].

### 2.5. RT-qPCR Validation

To verify the reliability of the transcriptome sequencing results, twelve differentially expressed genes were randomly selected for RT-qPCR validation. RNA extraction methods and RT-qPCR experimental procedures were conducted following the protocol described by An et al. [4]. Gene-specific primers were designed and synthesized by Shanghai Sangon Biotech Co., Ltd. (Table 1). Amplification was performed using the CFX96 Real-Time PCR System (Bio-Rad Laboratories, Inc., Hercules, CA, USA), with GAPDH serving as the internal reference gene. The relative gene expression levels were calculated using the 2^−ΔΔCt^ method, with each sample analyzed in triplicate.

### 2.6. Statistical Analysis

All experimental data are presented as mean ± standard error (Mean ± SE). Statistical analyses were conducted using GraphPad Prism 10 software, with group comparisons performed via the t-test. The significance levels were defined as follows: *p* < 0.05 indicates a significant difference, *p* < 0.01 indicates a highly significant difference, and *p* > 0.05 indicates no statistical difference.

## 3. Results

### 3.1. RNA Extraction Results

The RNA concentrations of the samples varied from 35 to 246 ng/μL, and the RIN values ranged from 5 to 7.3. All samples exhibited 260/280 ratios between 2.0 and 2.1, indicating the RNA’s high purity.

### 3.2. Sequencing Data Quality Assessment

Transcriptome sequencing was conducted on 12 samples, generating a total of 920,638,370 base pairs (bp) of raw reads. After filtering, 893,253,970 bp of high-quality clean reads were obtained, with Q30 bases comprising over 96.29% and GC content approximately 50.91%. These metrics indicate a high quality of sequencing data, suitable for subsequent in-depth analysis (see Table S1). Alignment results with the horse reference genome (EquCab3.0) (see Table S2) demonstrated that all samples achieved unique mapping rates exceeding 96%, while multi-mapped rates remained below 10%, thus confirming reliable alignment results for further analysis. The distribution of sequencing reads across exons, introns, and intergenic regions is illustrated in Figure S1. The highest proportion of reads was mapped to exons, suggesting that the sequencing data primarily originated from mature mRNA transcripts, as exons are the main regions encoding proteins. Inter-sample correlation analysis indicated that the Pearson correlation coefficient R^2^ between biological replicate samples was greater than 0.86, reflecting good experimental reproducibility and reasonable grouping (see Figure S2). Additionally, statistical analysis was performed on the FPKM values of each sample (see Figure S3 and Table S3). The results revealed that the distribution characteristics of log10(FPKM) values in each sample group were consistent with the size distribution of genes (see Figure S4).

### 3.3. Differentially Expressed Gene Analysis

**Figure 2:**
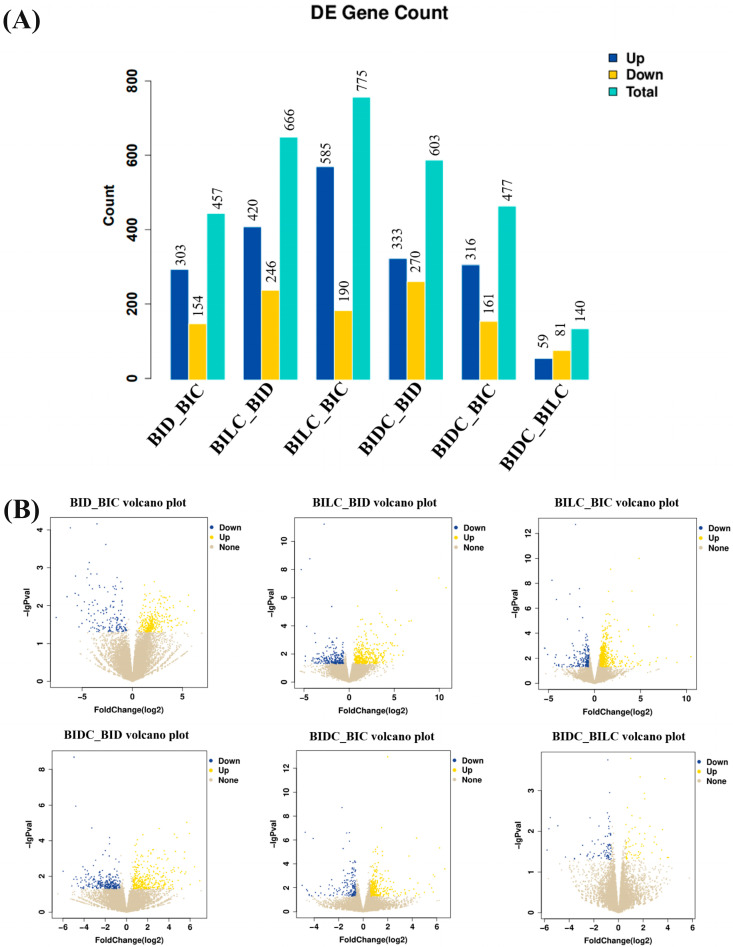
Illustrates the statistics of differentially expressed genes (**A**) alongside a volcano plot (**B**). Note: The horizontal axis represents the fold change in gene expression across various samples, while the vertical axis indicates the statistical significance of changes in expression levels. Each dot corresponds to a gene, with blue indicating up-regulated genes, yellow indicating down-regulated genes, and brown representing genes that are not significantly differentially expressed. The regions are defined as follows: BIDC refers to the dark-marked shoulder region; BILC refers to the light-marked shoulder region; BID denotes the dorsal midline; and BIC indicates the croup region.

The statistical results of differentially expressed genes across the six comparison groups are presented in Figure 2A. A varying number of differentially expressed genes were identified in each group, with the specific counts as follows: the BID vs. BIC group exhibited 457 differentially expressed genes; the BILC vs. BID group presented 666; the BILC vs. BIC group revealed 775; the BIDC vs. BID group showed 603; the BIDC vs. BIC group contained 477; and the BIDC vs. BILC group had 140. The volcano plot (Figure 2B) illustrates gene expression patterns by displaying the counts of up-regulated, down-regulated, and non-significantly changed genes.

### 3.4. GO Functional Enrichment Analysis

**Figure 3:**
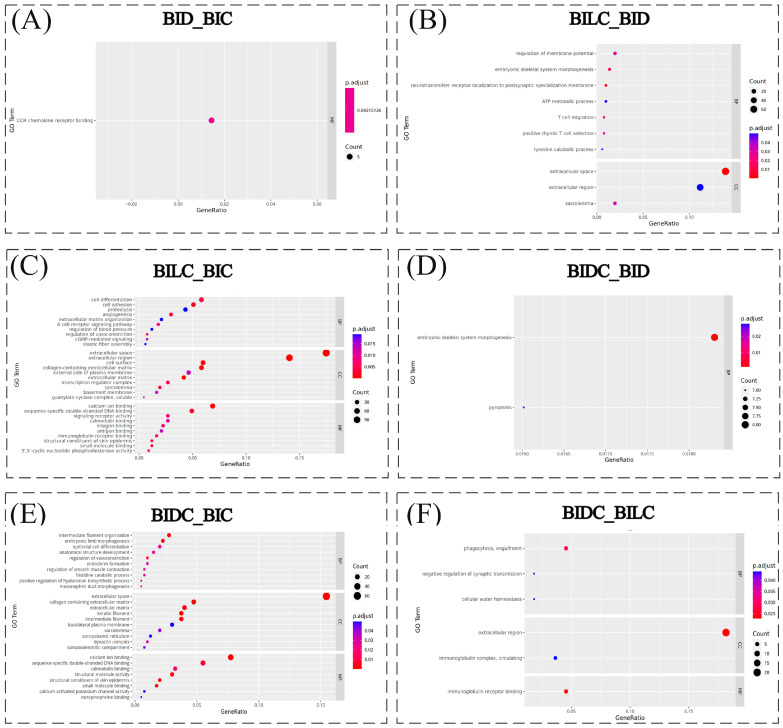
GO enrichment Bubble Plot. Note: The vertical axis represents the Gene Ontology (GO) terms, while the horizontal axis denotes the proportion of genes enriched in each term relative to the total number of genes. The color gradient indicates the adjusted *p*-values, with redder hues signifying more significant results. Additionally, the size of the bubbles corresponds to the number of genes enriched in each term, where larger bubbles indicate a greater number of genes. The regions are defined as follows: BIDC refers to the dark-marked shoulder region; BILC refers to the light-marked shoulder region; BID denotes the dorsal midline; and BIC indicates the croup region.

GO enrichment analysis of differentially expressed genes between the BID and BIC tissue comparison groups revealed only one statistically significant MF enrichment module (*p* < 0.05), specifically CCR family chemokine receptor binding (Figure 3A). In the functional enrichment analysis of the BILC and BID tissue comparison groups, ten significantly associated GO functional modules were identified. These categories encompass developmental regulation, metabolic processes, and extracellular microenvironment construction, including neurotransmitter receptor localization to the postsynaptic specialization membrane, embryonic skeletal system morphogenesis, regulation of membrane potential, T cell migration, and positive thymic T cell selection. At the metabolic level, the relevant processes include the ATP metabolic process and tyrosine catabolic process. Regarding cellular components, the identified modules include extracellular space, sarcolemma, and extracellular region (Figure 3B). In the GO functional enrichment analysis comparing the BILC and BIC organization groups, 61 significantly associated functional modules were detected, with their functional clustering exhibiting distinct structural biological characteristics. The main functional categories closely related to pigmentation, skin, and hair follicle development were subsequently screened, including cGMP-mediated signaling, positive regulation of phosphatidylinositol 3-kinase activity, calmodulin binding, structural constituent of skin epidermis (GO:0030280), basement membrane, extracellular matrix, calcium ion binding, cell surface, embryonic limb morphogenesis, mesenchyme migration, transcription regulator complex, and sequence-specific double-stranded DNA binding, among others (Figure 3C). In the comparison between BIDC and BID tissue groups, two significantly associated functional modules were identified, corresponding to embryonic skeletal system morphogenesis and pyroptosis within the BP category (Figure 3D). In the GO functional enrichment analysis of the BIDC versus BIC tissue comparison, a total of 42 significantly associated functional modules were identified. Notably, entries related to pigment regulation and skin hair follicle development were selected, including phosphatidylinositol-mediated signaling, positive regulation of hyaluronan biosynthetic process, calcium ion binding, structural constituent of skin epidermis, keratinization, intermediate filament organization, keratin filament, basolateral plasma membrane, embryonic limb morphogenesis, epithelial cell differentiation, and anatomical structure development, among others (Figure 3E). In the functional enrichment analysis comparing the BIDC and BILC groups, six significantly associated functional modules were detected, with functional clustering emphasizing immune regulation, metabolic homeostasis, and intercellular interactions. Among these, the immune regulation-related functions included phagocytosis, immunoglobulin complex circulation, and immunoglobulin receptor binding. Metabolism is associated with homeostasis maintenance, specifically cellular water homeostasis at the cellular level. Structural and signal regulation are linked to the extracellular region and negative regulation of synaptic transmission, among others (Figure 3F).

**Figure 4:**
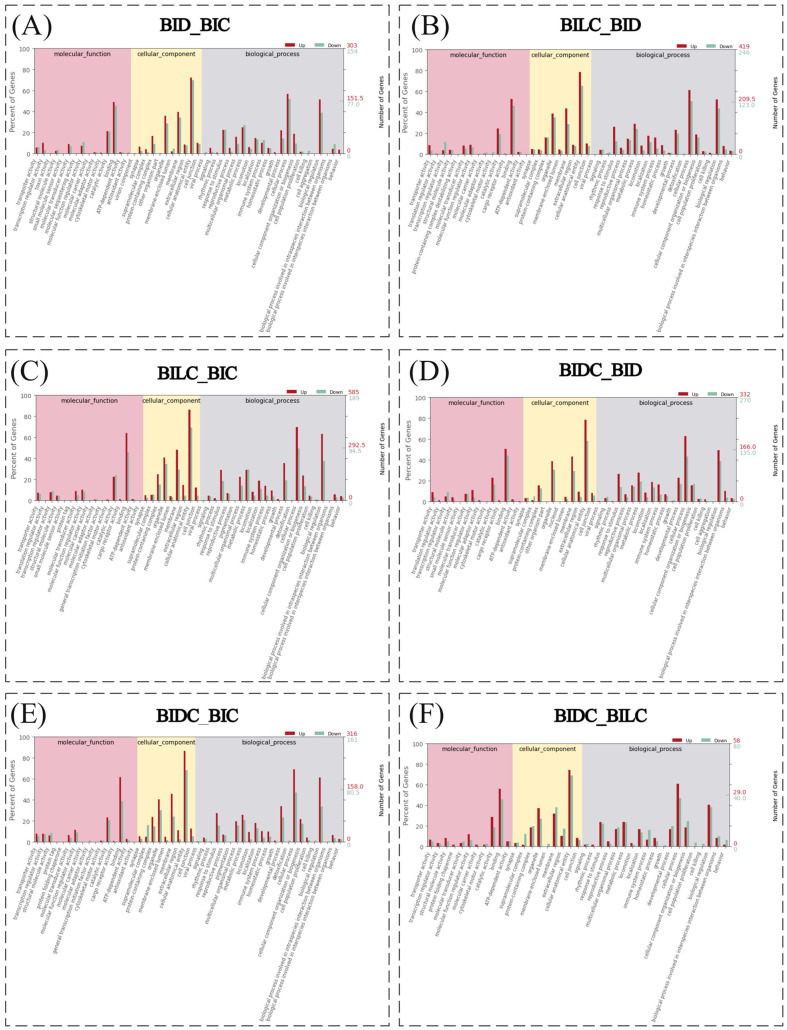
This illustrates the secondary classification of Gene Ontology (GO) enrichment analysis for significantly enriched differentially expressed genes. Note: The horizontal axis represents the secondary GO terms associated with the annotation results of these genes. The left vertical axis indicates the proportion of up-regulated and down-regulated differentially expressed genes, while the right vertical axis displays the absolute numbers of up-regulated and down-regulated differentially expressed genes. The regions are defined as follows: BIDC refers to the dark-marked shoulder region; BILC refers to the light-marked shoulder region; BID denotes the dorsal midline; and BIC indicates the croup region.

To visually display the GO statistical results of differentially expressed gene sets, we selected the top 50 significantly enriched terms (*p* < 0.05) to generate the accompanying bar chart. The GO statistical bar chart results for differentially expressed genes across six comparison groups reveal that the top 10 GO terms with the highest number of differentially expressed genes in the BID and BIC comparison groups are as follows: cellular anatomical entity (CC, 220 up-regulated, 108 down-regulated), cellular process (BP, 172 up-regulated, 80 down-regulated), binding (MF, 156 up-regulated, 60 down-regulated), biological regulation (BP, 149 up-regulated, 70 down-regulated), membrane (CC, 121 up-regulated, 53 down-regulated), organelle (CC, 109 up-regulated, 44 down-regulated), metabolic process (BP, 76 up-regulated, 42 down-regulated), response to stimulus (BP, 68 up-regulated, 35 down-regulated), developmental process (BP, 67 up-regulated, 22 down-regulated), and catalytic activity (MF, 65 up-regulated, 32 down-regulated). Additional GO terms related to biological processes, cellular components, and molecular functions are also included (Figure 4A, Table S4). In the BILC and BID comparison groups, the top 10 GO terms with the highest number of differentially expressed genes are: cellular anatomical entity (CC, 330 up-regulated, 161 down-regulated), cellular process (BP, 257 up-regulated, 125 down-regulated), binding (MF, 221 up-regulated, 114 down-regulated), biological regulation (BP, 220 up-regulated, 108 down-regulated), membrane (CC, 184 up-regulated, 71 down-regulated), organelle (CC, 163 up-regulated, 87 down-regulated), metabolic process (BP, 123 up-regulated, 60 down-regulated), response to stimulus (BP, 111 up-regulated, 31 down-regulated), catalytic activity (MF, 103 up-regulated, 48 down-regulated), and developmental process (BP, 99 up-regulated, 49 down-regulated), along with other relevant biological processes, cellular components, and molecular function GO terms (Figure 4B, Table S5). The ten GO terms exhibiting the highest numbers of differentially expressed genes in the comparisons between BILC and BIC groups were as follows: cellular anatomical entity (CC) with 505 up-regulated and 132 down-regulated genes; cellular process (BP) with 409 up-regulated and 94 down-regulated genes; binding (MF) with 375 up-regulated and 87 down-regulated genes; biological regulation (BP) with 372 up-regulated and 72 down-regulated genes; membrane (CC) with 283 up-regulated and 56 down-regulated genes; organelle (CC) with 240 up-regulated and 66 down-regulated genes; developmental process (BP) with 209 up-regulated and 66 down-regulated genes; response to stimulus (BP) with 170 up-regulated and 35 down-regulated genes; metabolic process (BP) with 170 up-regulated and 56 down-regulated genes; and protein-containing complex (CC) with 146 up-regulated and 29 down-regulated genes. These findings are illustrated in Figure 4C and detailed in Table S6. Similarly, the ten GO terms with the highest numbers of differentially expressed genes in the comparisons between BIDC and BID groups were: cellular anatomical entity (CC) with 261 up-regulated and 158 down-regulated genes; cellular process (BP) with 210 up-regulated and 117 down-regulated genes; binding (MF) with 169 up-regulated and 119 down-regulated genes; biological regulation (BP) with 165 up-regulated and 105 down-regulated genes; membrane (CC) with 144 up-regulated and 80 down-regulated genes; organelle (CC) with 129 up-regulated and 83 down-regulated genes; metabolic process (BP) with 93 up-regulated and 52 down-regulated genes; response to stimulus (BP) with 89 up-regulated and 38 down-regulated genes; catalytic activity (MF) with 77 up-regulated and 44 down-regulated genes; and developmental process (BP) with 77 up-regulated and 46 down-regulated genes. These results are depicted in Figure 4D and further elaborated in Table S7. The top ten GO terms with the highest number of differentially expressed genes in the BIDC and BIC comparison groups were as follows: cellular anatomical entity (CC), with 274 up-regulated and 110 down-regulated genes; cellular process (BP), with 218 up-regulated and 76 down-regulated genes; binding (MF), with 195 up-regulated and 63 down-regulated genes; biological regulation (BP), with 194 up-regulated and 54 down-regulated genes; membrane (CC), with 145 up-regulated and 39 down-regulated genes; organelle (CC), with 128 up-regulated and 49 down-regulated genes; developmental process (BP), with 107 up-regulated and 38 down-regulated genes; response to stimulus (BP), with 87 up-regulated and 25 down-regulated genes; metabolic process (BP), with 82 up-regulated and 34 down-regulated genes; and protein-containing complex (CC), and with 75 up-regulated and 24 down-regulated genes. These terms represent various biological processes, cellular components, and molecular functions (Figure 4E, Table S8). In the BIDC and BILC comparison groups, the top ten GO terms with the highest number of differentially expressed genes were: cellular anatomical entity (CC), with 44 up-regulated and 56 down-regulated genes; cellular process (BP), with 36 up-regulated and 38 down-regulated genes; binding (MF), with 33 up-regulated and 37 down-regulated genes; biological regulation (BP), with 24 up-regulated and 31 down-regulated genes; organelle (CC), with 22 up-regulated and 22 down-regulated genes; membrane (CC), with 19 up-regulated and 31 down-regulated genes; catalytic activity (MF), with 17 up-regulated and 15 down-regulated genes; response to stimulus (BP), with 14 up-regulated and 18 down-regulated genes; metabolic process (BP), with 14 up-regulated and 19 down-regulated genes; and protein-containing complex (CC), with 11 up-regulated and 16 down-regulated genes. These terms also encompass various biological processes, cellular components, and molecular functions (Figure 4F, Table S9).

### 3.5. KEGG Pathway Enrichment Analysis

**Figure 5:**
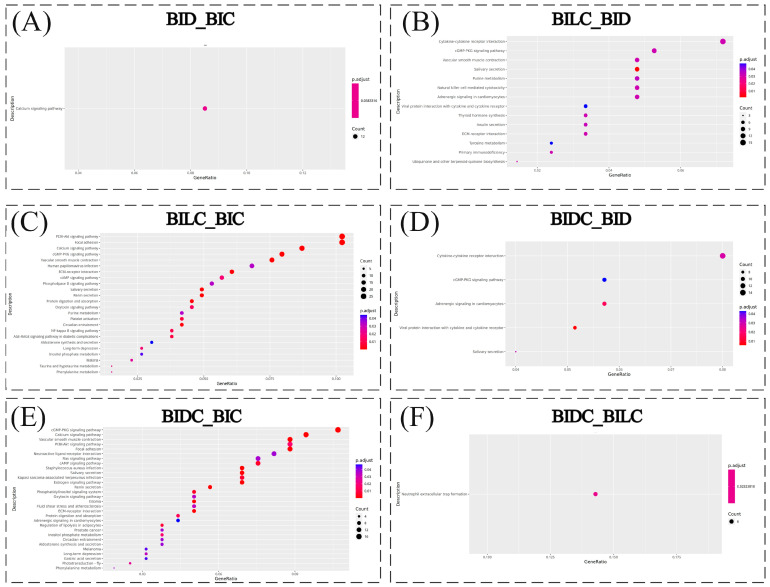
KEGG Enrichment Bubble Plot. Note: The vertical axis represents the KEGG pathways, while the horizontal axis illustrates the proportion of genes enriched in each pathway relative to the total number of genes. The color indicates the adjusted *p*-value (with redder colors representing higher significance), and the size of the bubbles corresponds to the number of genes enriched in the respective pathway (larger bubbles indicate a greater number of genes). The regions are defined as follows: BIDC refers to the dark-marked shoulder region; BILC refers to the light-marked shoulder region; BID denotes the dorsal midline; and BIC indicates the croup region.

Through KEGG pathway enrichment analysis, a total of 252 signaling pathways were identified. The top 30 most significant pathways, or all pathways if fewer than 30 were found, were illustrated using bubble plots. Under the condition of *p* < 0.05, the KEGG pathway enrichment analysis indicated that a single calcium signaling pathway (Calcium signaling pathway, map04020) was significantly enriched in the BID and BIC comparison groups. This calcium signaling pathway (map04020) (refer to Figure S5) was enriched with 12 genes, including CAMK1, AGTR1, PDE1A, FGF10, ADCY2, PDGFB, GRPR, CYSLTR1, HTR7, ADCY1, PDE1C, and CHRM2, as shown in Figure 5A. In the KEGG pathway enrichment analysis comparing the BILC and BID groups, 279 signaling pathways were identified, among which 14 pathways showed significant enrichment. These included Tyrosine metabolism, ECM-receptor interaction, Cytokine-cytokine receptor interaction, and Viral protein interaction with cytokine and cytokine receptor. Four genes, namely HPD, HGD, MAOB, and TAT, were enriched in the tyrosine metabolism pathway (map00350) related to pigmentation (Figure S6) (Figure 5B). In the KEGG pathway enrichment analysis comparing the BILC and BIC groups, a total of 287 pathways were identified, from which the top 30 most differentially expressed pathways were selected for visualization in a bubble chart. Significant enrichment was found in 24 pathways, including Focal Adhesion, ECM-Receptor Interaction, cAMP Signaling Pathway, Calcium Signaling Pathway, PI3K-Akt Signaling Pathway, and Phenylalanine Metabolism, among others. In the cAMP signaling pathway (map04024) (see Figure S7), 15 genes were found to be enriched: PDE3B, PLN, ADRB2, ATP2B2, OXTR, CACNA1D, PDE4C, JUN, MYL9, EDN3, GRIN1, PLCE1, ATP1A2, VAV3, and PDE3A. Similarly, in the calcium signaling pathway (map04020) (see Figure S8), 23 genes were enriched, namely: NOS1, PLN, CACNA1G, RYR3, FLT4, PDGFRB, ADRB2, ATP2B2, CAMK1, OXTR, CACNA1D, AGTR1, PDE1A, MYLK, PLCB1, NTRK2, GRIN1, PDGFB, GRPR, EGF, PLCE1, HGF, and AVPR1A (refer to Figure 5C). In the KEGG pathway enrichment analysis comparing the BIDC group with the BID control group, a total of 261 pathways were co-enriched. The top 30 most differentially enriched pathways were selected to generate a bubble plot, which primarily involved cytokine-cytokine receptor interactions, viral protein interactions with cytokines and cytokine receptors, and the cGMP-PKG signaling pathway (Figure 5D). In the comparison between the BIDC and BIC groups, a total of 251 pathways were co-enriched. The top 30 differentially expressed pathways were selected to generate bubble charts, revealing significant enrichment in focal adhesion, the calcium signaling pathway, ECM-receptor interaction, the cAMP signaling pathway, the PI3K-Akt signaling pathway, and melanoma. In the melanoma pathway (Melanoma, map05218) (Figure S9), five genes were enriched (PDGFRB, GADD45G, PDGFB, PIK3CD, EGF). In the calcium signaling pathway (map04020) (Figure S10), 14 genes were enriched (NOS1, PDGFRB, ADRB2, ATP2B2, CAMK1, OXTR, ITPR1, PDE1A, NTRK2, PDGFB, LOC100070422, CALML6, EGF, PLCE1, PTGER3). In the cAMP signaling pathway (map04024) (Figure S11), 12 genes were enriched (PDE3B, ADRB2, ATP2B2, OXTR, MYL9, EDN3, LOC100070422, PIK3CD, CALML6, PLCE1, ATP1A2, PTGER3) (Figure 5E). A total of 132 pathways were enriched in the BIDC versus BILC comparison group, with significant enrichment observed in neutrophil extracellular trap formation (Figure 5F).

### 3.6. RT-qPCR Validation Results

**Figure 6:**
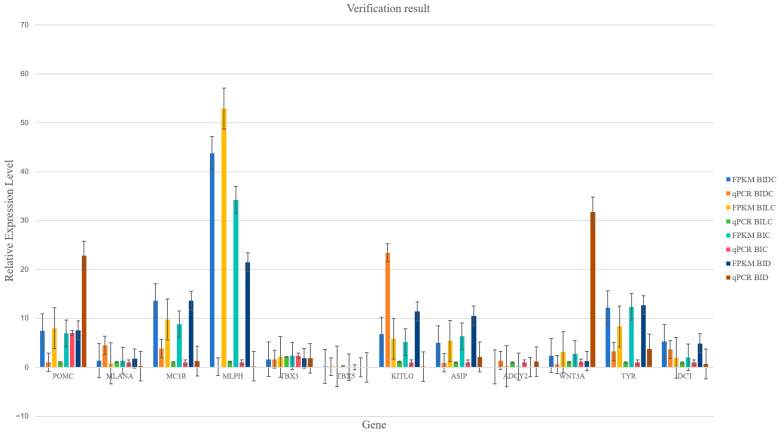
Comparison of the RNA-seq result with qPCR. Ten different genes were randomly selected, and the results were converted to log2 (fold change).

A total of twelve randomly selected differentially expressed genes (DEGs) were utilized to validate the results obtained from RNA sequencing. The RT-qPCR findings corroborated the reliability of the RNA sequencing data, as illustrated in Figure 6 (Table S10).

## 4. Discussion

The distinctive “Bider” markings of the Dun Mongolian horse, characterized by their irregular yet symmetrical patterns, provide a unique model for studying the spatial regulation of skin pigmentation in mammals. This study presents the first comprehensive transcriptomic comparison between the Bider marking region and the dorsal midline/croup skin regions, revealing a complex, region-specific gene expression profile. The central finding demonstrates that the formation of this striking pattern is not controlled by a single gene; rather, it is orchestrated by a coordinated network involving multiple biological processes, with calcium signaling and cAMP signaling pathways emerging as core regulatory factors.

The most compelling evidence from KEGG analysis underscores the pivotal role of the calcium signaling pathway. This pathway was significantly enriched in the comparison groups using the buttock (BIC) as the reference tissue (e.g., BID vs. BIC, BILC vs. BIC, and BIDC vs. BIC), indicating its fundamental role in defining regional skin characteristics. Calcium ions, as ubiquitous secondary messengers [29], are known to regulate the activity of key transcription factors, such as MITF in melanocytes, with MITF controlling the expression of tyrosinase and other melanogenic enzymes [30]. This pathway exhibited constitutive enrichment across multiple comparison groups, suggesting that differential calcium signaling may shape a permissive microenvironment in predetermined dark skin regions, priming melanocytes into a pre-adapted state to meet higher functional demands [31]. Concurrently, the enrichment of the cAMP signaling pathway, particularly in the BIDC versus BIC comparison, further consolidates the role of this classical melanocyte-stimulating pathway [32]. Activated through α-MSH/MC1R and mediated by the cAMP/PKA/CREB signaling cascade, this canonical pathway upregulates the expression of the master regulator MITF, thereby driving melanin synthesis [33]. The presence of the EDN3 (endothelin 3) gene in this enriched pathway is noteworthy, as EDN3 serves as a potent mitogen and chemokine for melanocyte precursors [34]. In the same comparison group, the co-enrichment of the melanoma pathway (involving genes such as PDGFB and PIK3CD) strongly suggests the maintenance of a robust signaling network that supports melanocyte proliferation and survival. PDGFB is known to be a key regulator of melanocyte proliferation and differentiation [35], while signaling pathways such as PI3K/AKT constitute the core network governing melanocyte proliferation, survival, and differentiation [36]. In melanoma, the PI3K/AKT pathway is commonly activated to promote tumor cell proliferation and survival [37]. Multiple growth factor signals, such as PDGF and HGF, act synergistically through downstream pathways to collectively establish a network that supports cell growth [38]. Additionally, the direct enrichment of the tyrosine metabolic pathway in the comparison between BILC and BID establishes a direct link between the observed transcriptional differences and the core biochemical process of melanin synthesis, with tyrosine serving as the primary substrate [32].

In addition to specific pigment-related pathways, the Gene Ontology (GO) analysis in this study provides crucial insights into the developmental and structural basis of pattern formation. When compared to the buttock (BIC), the significant enrichment of terms such as “embryonic limb morphogenesis,” “anatomical structure development,” and “epidermal structure” in the comparative groups involving BILC and BIDC offers valuable information. This suggests that the skin constituting the Bider marking region possesses distinct structural characteristics compared to the more posterior buttock skin. The enrichment of pathways related to “extracellular matrix” and “focal adhesion” supports the notion that regional differences exist in the dermal microenvironment—the “soil” where melanocytes reside [39,40]. The specialized extracellular matrix can influence the migration, adhesion, and capacity of melanocytes to receive mitotic signals [41]. Therefore, the formation of Bider marking may not solely originate from intrinsic differences in melanocytes but is more likely guided by a pre-patterned skin “topography” that instructs pigment cell behavior. Interestingly, comparative analysis between two adjacent shoulder regions (BIDC and BILC) revealed an enrichment of immunity-related terms such as “phagocytosis” and “immunoglobulin complex.” This suggests that local immune surveillance or regulation may play a potential role in maintaining sharp boundaries between dark and light stripes [42], possibly through interactions with the hair follicle melanin unit [43].

Integrating these findings, we propose a multi-level model for the ontogeny of Bider markings. Initially, during embryonic development, positional signals establish distinct identities in different shoulder skin regions, involving genes related to limb morphogenesis and extracellular matrix composition. In the dark stripe (BIDC) region, this creates a microenvironment conducive to melanocyte function, characterized by enhanced endothelin and/or other receptor signaling. This activation triggers downstream calcium and cAMP second messenger systems, stabilizes MITF, and drives the expression of melanogenic enzymes, as reflected in tyrosine metabolism. Concurrently, pathways such as PI3K-Akt and focal adhesion support melanocyte survival and their integration within the skin. The adjacent light-colored area (BILC), although sharing a similar developmental origin with BIDC, may lack or suppress these activating signals, potentially through local immune regulation, thereby resulting in a hypopigmented phenotype. The buttock (BIC) represents a developmentally more distant skin region with a fundamentally distinct transcriptomic background.

We acknowledge the limitations of this study. While the sample size was adequate for exploratory transcriptome research, validation in larger populations is necessary. Additionally, bulk tissue RNA sequencing averages signals across all cell types, meaning the observed expression changes may originate from melanocytes, keratinocytes, fibroblasts, or immune cells. The definitive cellular sources of key signals and the causal relationships within the proposed network require functional validation. Future studies utilizing single-cell RNA sequencing and spatial transcriptomics will be crucial for resolving cell type-specific contributions and precisely localizing signaling hubs. Furthermore, functional experiments are necessary, such as regulating candidate genes (EDN3, PIK3CD) in melanocyte or skin explant cultures, to verify their necessity and sufficiency in driving pigment pattern differences.

The reliability of our RNA-seq data was assessed by qPCR validation of 12 differentially expressed genes across four comparison groups. While the overall expression trends between light and dark skin regions were largely consistent between the two methodologies, notable discrepancies in absolute expression levels were observed for several genes, most prominently POMC, WNT3A, and MLPH. For instance, in the BID comparison, the qPCR value for POMC was substantially higher than its FPKM value, whereas for MLPH in the BIDC group, the FPKM value significantly exceeded its qPCR measurement. Such quantitative differences between RNA-seq (FPKM) and qPCR are not uncommon and can be attributed to several inherent technical factors. These include differences in dynamic range, normalization procedures (global transcriptome normalization vs. reference gene normalization) [44], and primer amplification efficiencies in qPCR [45]. Furthermore, the moderate RNA integrity (RIN 5-7.3) of some samples could have differentially impacted the representation of full-length transcripts in the RNA-seq library compared to the targeted qPCR assays [46]. Although this validation confirms the directional change in gene expression, the observed quantitative variances underscore the methodological variations between transcriptomic and PCR-based quantification [47].

## 5. Conclusions

This study conducted a transcriptome analysis of skin tissues from differentially pigmented regions of the Dun Mongolian horse’s Bider markings. The findings reveal that the formation of these markings is associated with the differential expression of numerous genes and is regulated by multiple key pigment-related pathways.

## Figures and Tables

**Table 1:** Primer sequence information for target genes and reference genes.

Primer Name	Primer Sequences (5′–3′)	Usage
GAPDH-F	GGCGATGCTGGTGCTGAATATG	reference genes
GAPDH-R	AGCAGAAGGAGCAGAGATGATGAC	
MC1R-F	CGCCAAGAACCGCAACCTG	target genes
MC1R-R	CCTCCAGCAGCAGCAAGATTG	
MLPH-F	ACGGATGAGGATGGAGAACTGG	
MLPH-R	CGGAGTGGAGTCGTCAGAGTC	
POMC-F	GTTCTCCTCCAGAGCCCACAC	
POMC-R	TTCTTCCTCTCTTTCTCCTCCTTCG	
MLANA-F	TACTGCTCATTGGCTGTTGGTATTG	
MLANA-R	CGTGGACATCTTCCTGTTAAGGTAC	
KITLG-F	GCATTGCCAGCATTCTTTTC	
KITLG-R	TGCCCTTGTAAGATTTGGTTG	
ASIP-F	TCACCTGAGGAGAAGCCCAAAG	
ASIP-R	TTCAATGCCATGATAGAGACAGAAGG	
TBX3 F	TCCTCCACGCTCTCCTCCAG	
TBX3 R	TCCAAGCCGCTGACCAACC	
TBX5 F	CCAACACTTCTCTGCTCACTTCAC	
TBX5 R	ACTGAGGTCTGGTGCTGGAAC	
ADCY2-F	ACTGTCTCCACCATCCATCCATC	
ADCY2-R	CTGCTTTCTCCGTGACCCATTC	
WNT3A-F	GCCATCGGTGACTTCCTCAA	
WNT3A-R	ACCTTGAAGTGGGTGTAGCG	
TYR-F	AGCCTGTGCCTCCTCCAAGAAC	
TYR-R	AGATGGTGCGTTGGACAGAATGAC	
DCT-F	ACGCCATTGACCTGCCAGTTG	
DCT-R	AACCAAAGCCACCAGCATTCCC	

## Data Availability

The transcriptome data generated in this study have been submitted to the NCBI database, Temporary Submission ID: SUB16077915. After the data are made public, the corresponding BioSample accession numbers will be: SAMN56695043 to SAMN56695054. The original contributions presented in the study are included in the article; further inquiries can be directed to the corresponding authors.

## Supplementary Materials

The following supporting information can be downloaded at: https://www.mdpi.com/article/10.3390/ani16081145/s1, Figure S1: Distribution of reads in different regions of the reference genome; Figure S2: mRNA sequencing correlation coefficient analysis between different samples; Figure S3: FPKM distribution; Figure S4: Box plot of FPKM; Figure S5: BID_BIC map04020; Figure S6: BILC_BID map00350; Figure S7: BILC_BIC map04024; Figure S8: BILC_BIC map04020; Figure S9: BIDC_BIC map05218; Figure S10: BIDC_BIC map04020; Figure S11: BIDC_BIC map04024; Table S1: Statistical analysis of filtered RNA-Seq data for Mongolian Bider horses; Table S2: Statistical analysis of reference sequence alignment for RNA-Seq data for Mongolian Bider horses; Table S3: Differentially Expressed Genes; Table S4: BID_BIC.GO enrichment analysis secondary classification Up_Down; Table S5: BILC_BID.GO enrichment analysis secondary classification Up_Down; Table S6: BILC_BIC.GO enrichment analysis secondary classification Up_Down; Table S7: BIDC_BID.GO enrichment analysis secondary classification Up_Down; Table S8: BIDC_BIC.GO enrichment analysis secondary classification Up_Down; Table S9: BIDC_BILC.GO enrichment analysis secondary classification Up_Down; Table S10: FPKMs and RT-qPCR data.

## Abbreviations

The following abbreviations are used in this manuscript:

BIDC	the dark-marked area on the shoulder
BILC	the light-marked area on the shoulder
BID	the dorsal midline
BIC	the croup
