# Supplementary figures and images for: Unraveling the Molecular Mechanism of Bider Marking Formation in Dun Mongolian Horses Through Transcriptome Sequencing
Source: Animals (Basel). 2026 Apr 9;16(8):1145. doi: 10.3390/ani16081145 (PMC13113289; doi:10.3390/ani16081145)

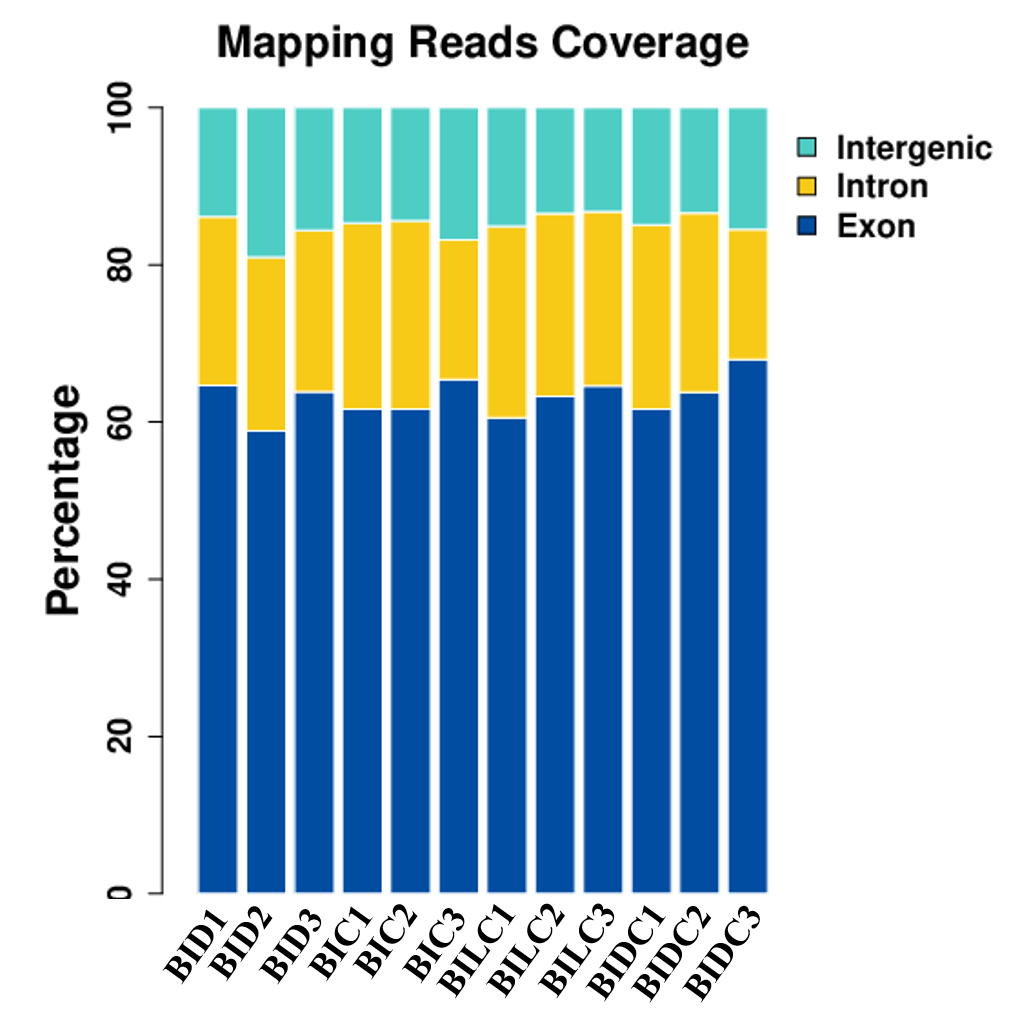

Supplement: Supplementary file 1 [file animals-16-01145-s001.zip › Figure S1 Distribution of reads in different regions of the reference genome.png]

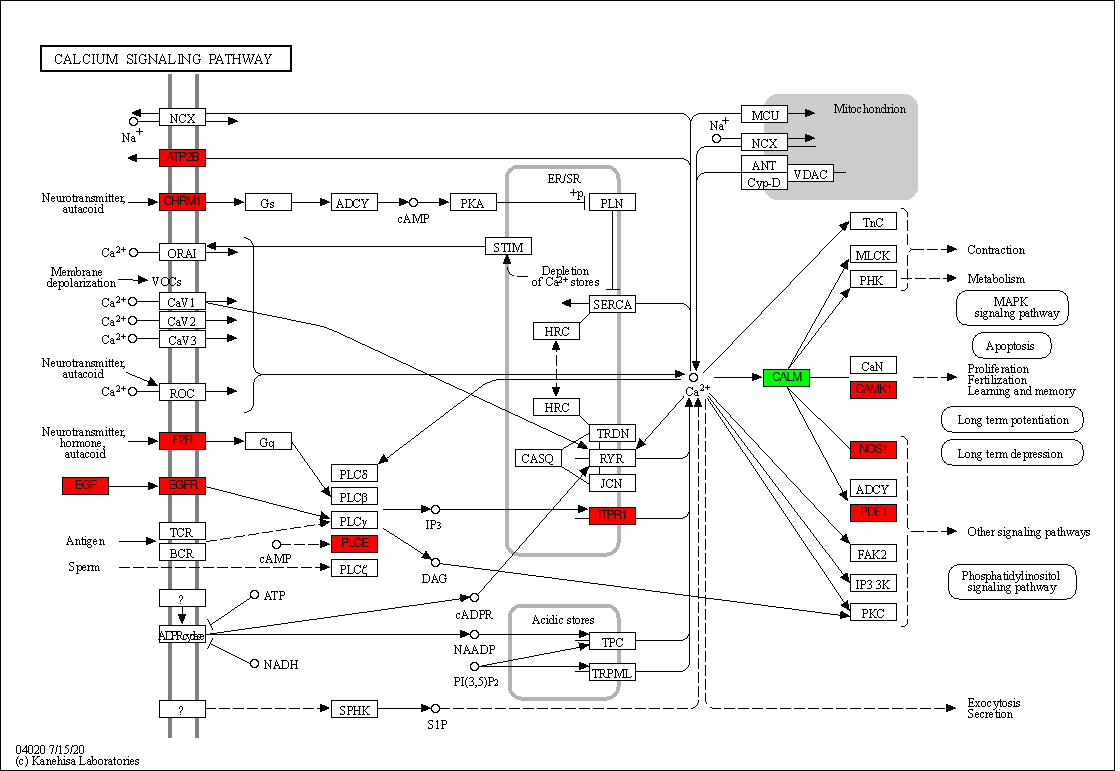

Supplement: Supplementary file 1 [file animals-16-01145-s001.zip › Figure S10 BIDC_BIC map04020.png]

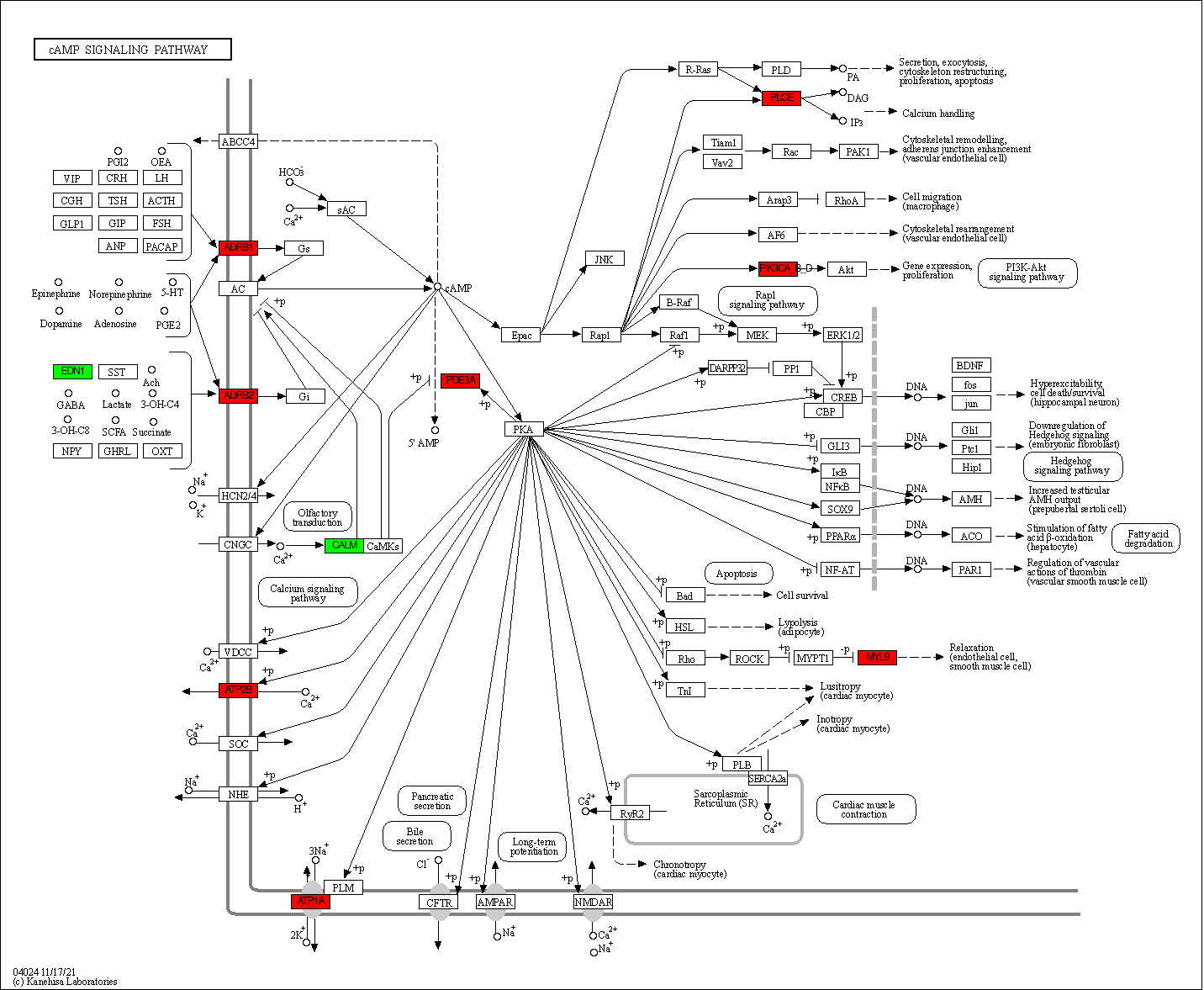

Supplement: Supplementary file 1 [file animals-16-01145-s001.zip › Figure S11 BIDC_BIC map04024.png]

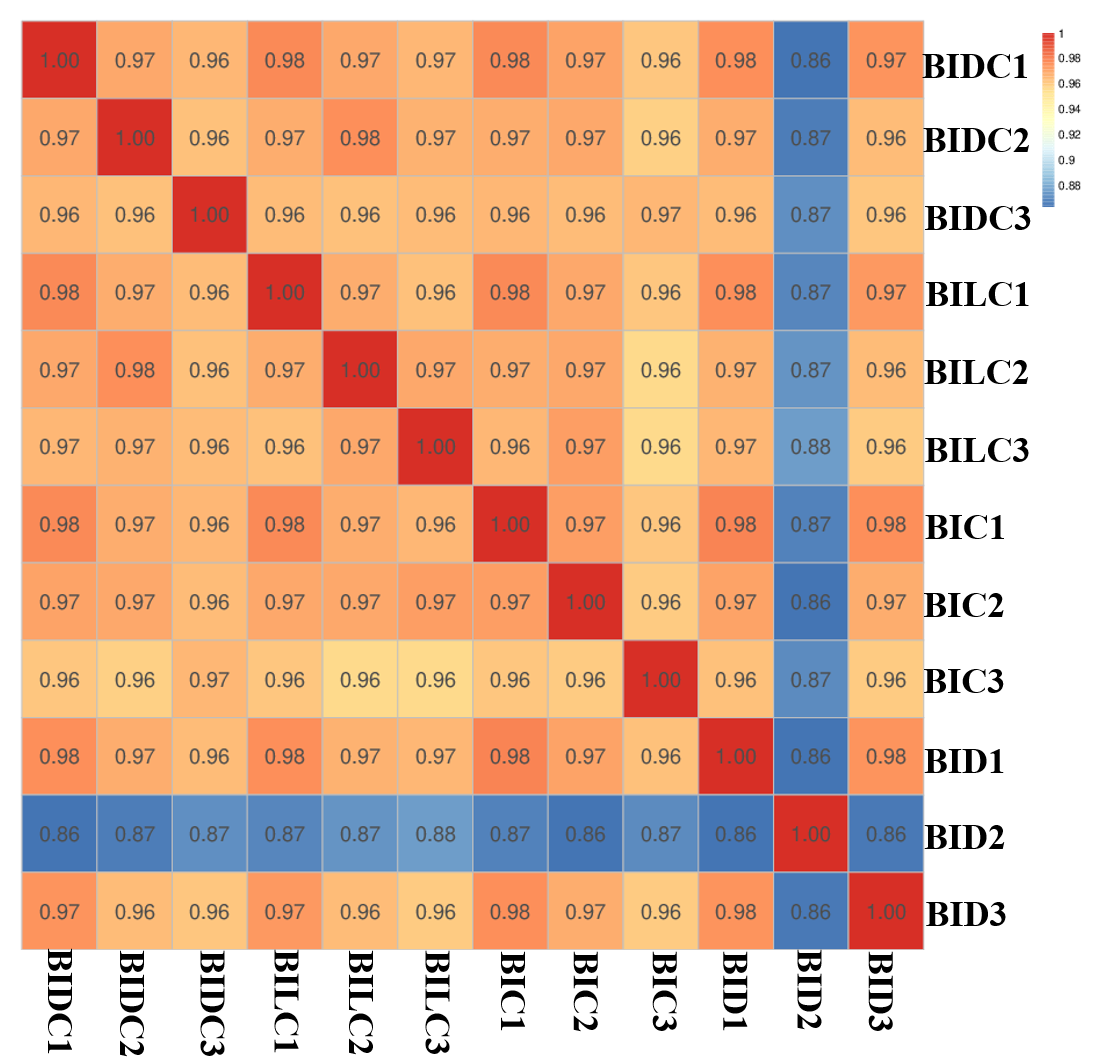

Supplement: Supplementary file 1 [file animals-16-01145-s001.zip › Figure S2 mRNA sequencing correlation coefficient analysis between different samples.png]

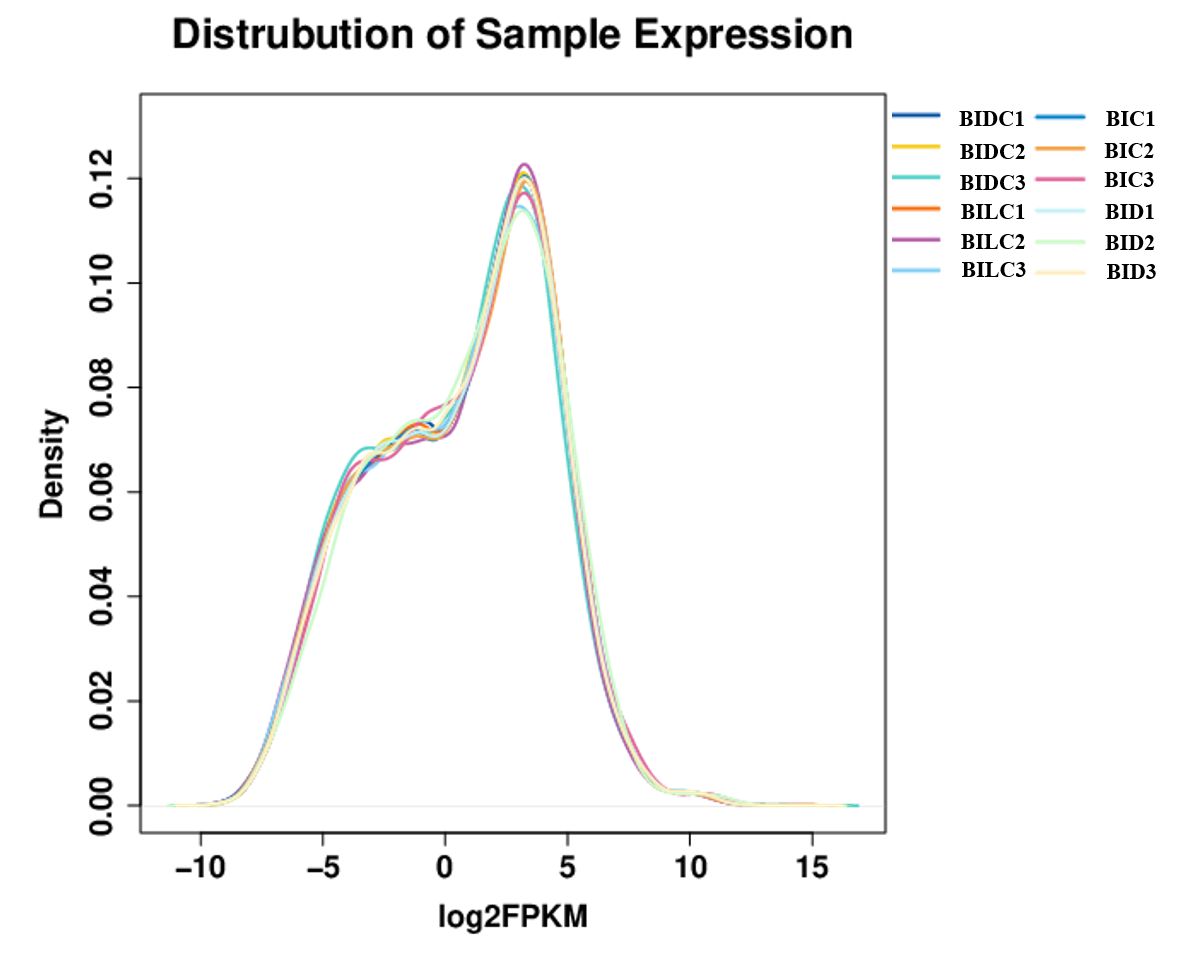

Supplement: Supplementary file 1 [file animals-16-01145-s001.zip › Figure S3 FPKM distribution.png]

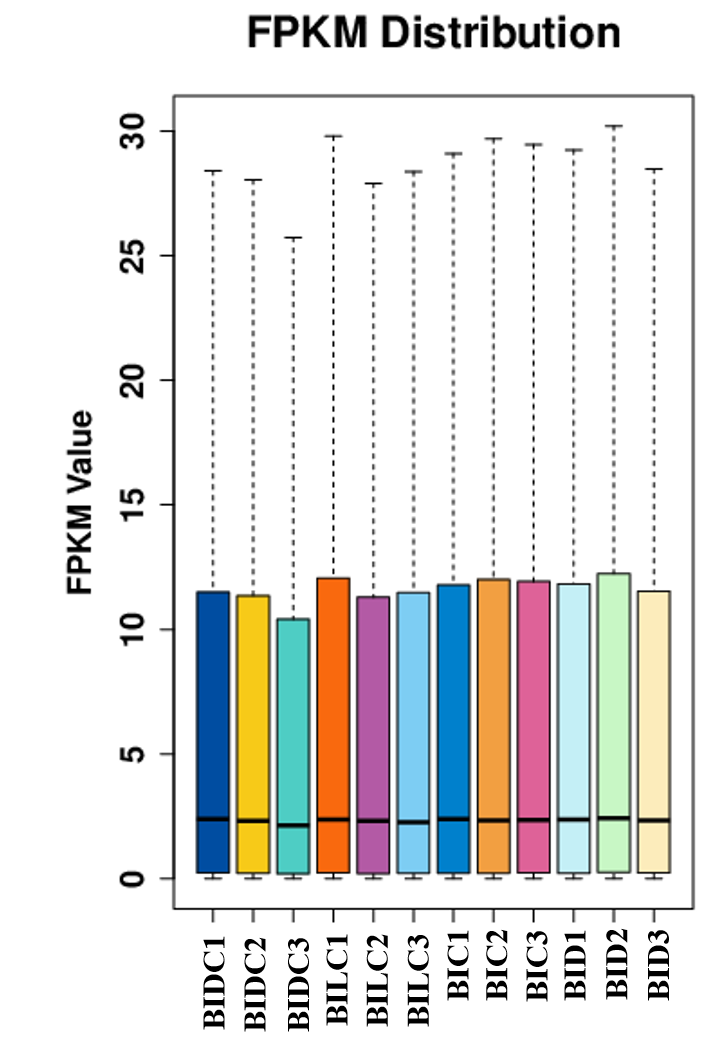

Supplement: Supplementary file 1 [file animals-16-01145-s001.zip › Figure S4 Box plot of FPKM.png]

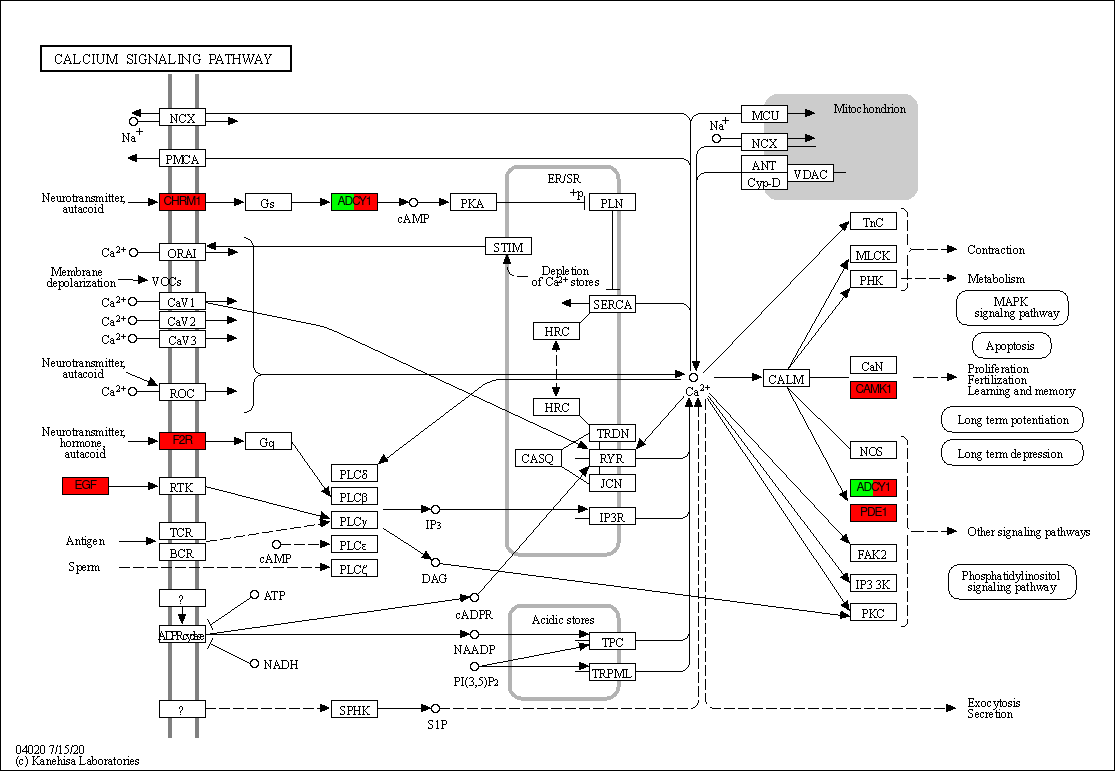

Supplement: Supplementary file 1 [file animals-16-01145-s001.zip › Figure S5 BID_BIC map04020.png]

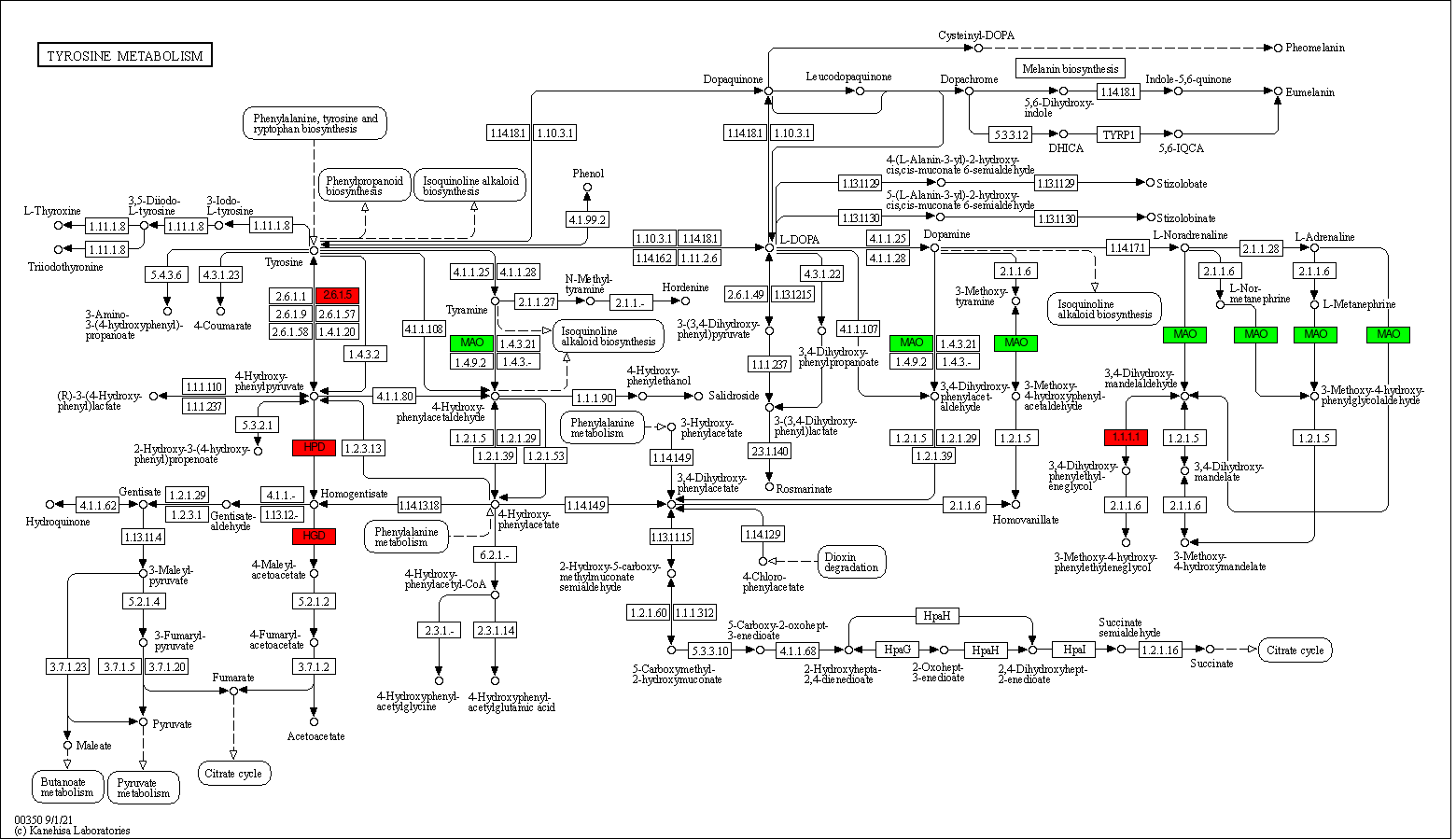

Supplement: Supplementary file 1 [file animals-16-01145-s001.zip › Figure S6 BILC_BID map00350.png]

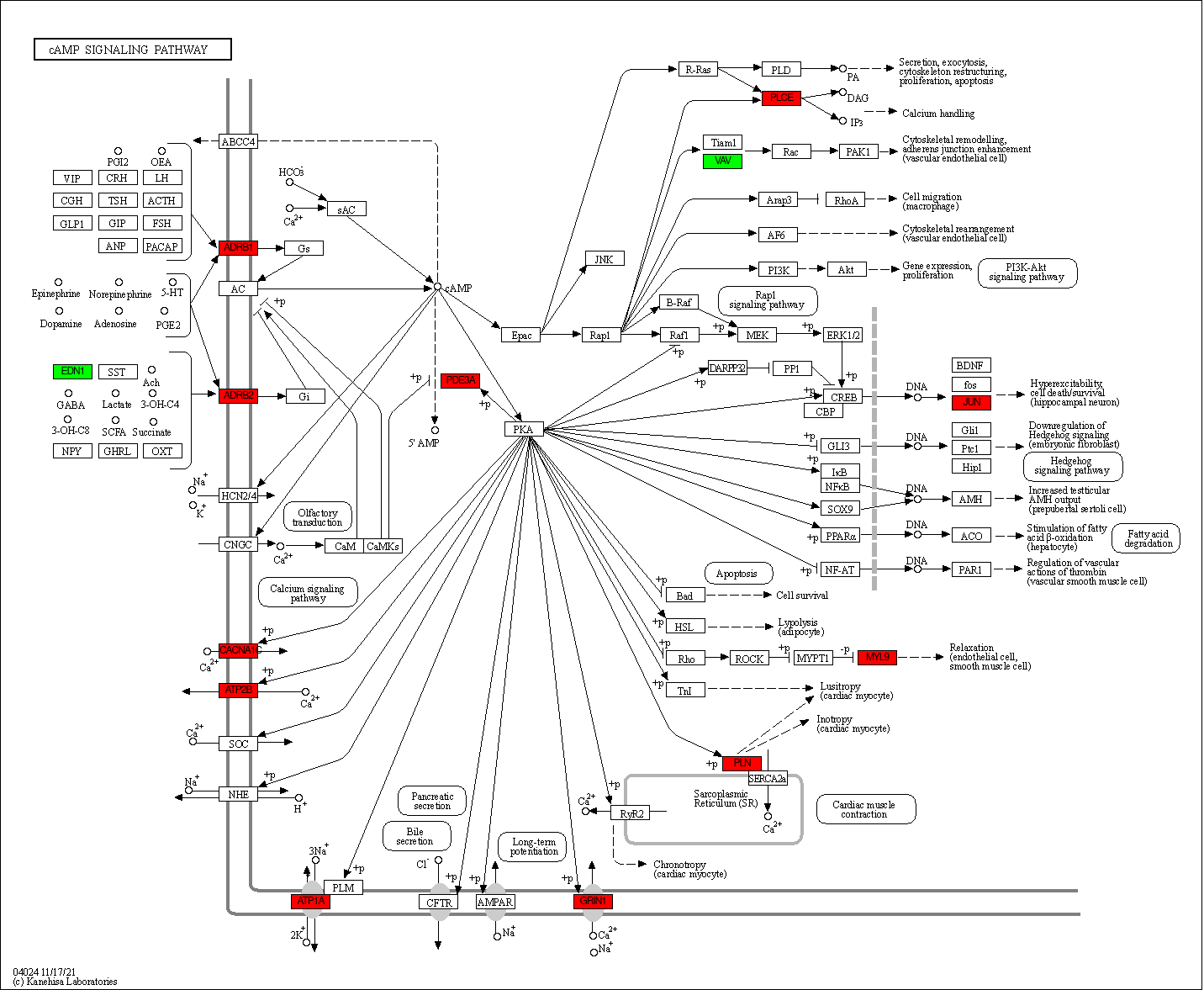

Supplement: Supplementary file 1 [file animals-16-01145-s001.zip › Figure S7 BILC_BIC map04024.png]

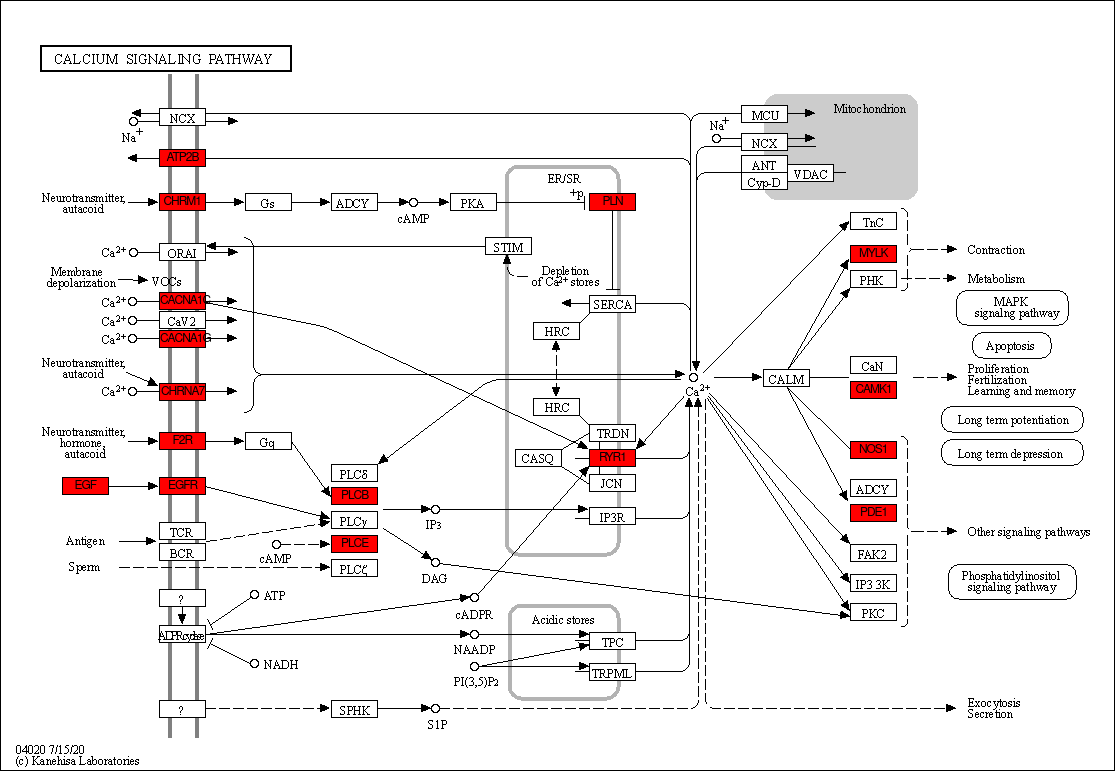

Supplement: Supplementary file 1 [file animals-16-01145-s001.zip › Figure S8 BILC_BIC map04020.png]

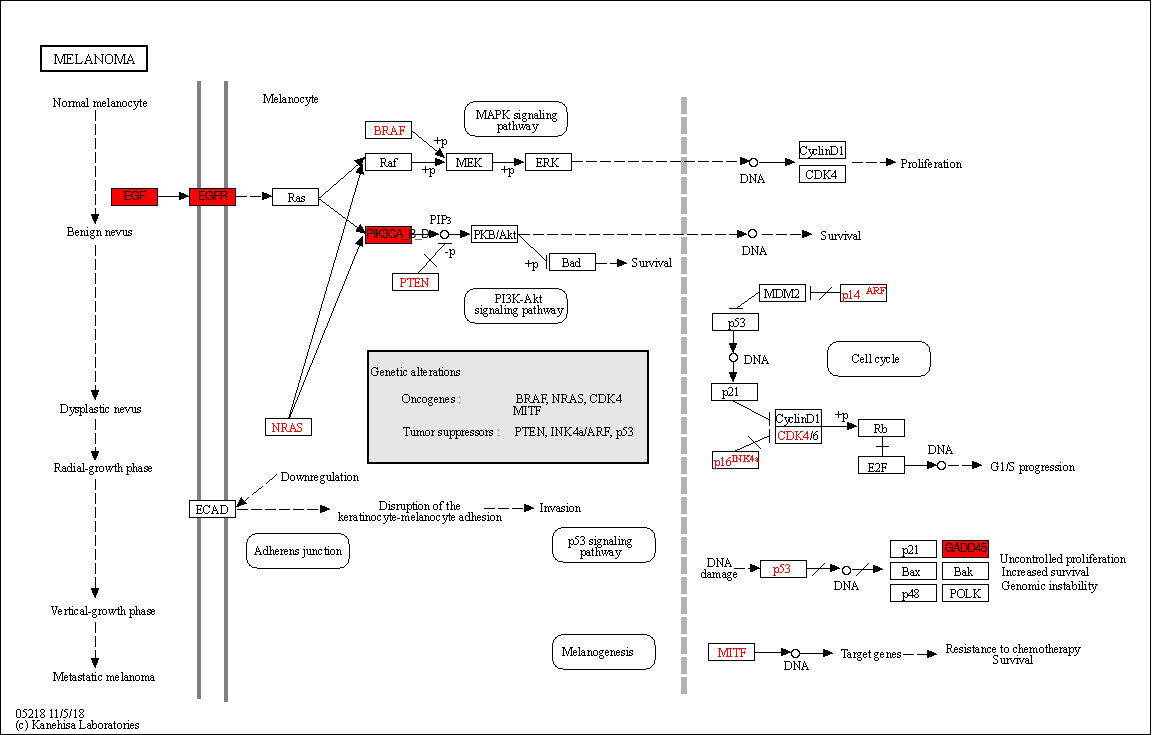

Supplement: Supplementary file 1 [file animals-16-01145-s001.zip › Figure S9 BIDC_BIC map05218.png]
